# Arthroscopic Removal of a Wire Fragment from the Posterior Septum of the Knee following Tension Band Wiring of a Patellar Fracture

**DOI:** 10.1155/2015/827140

**Published:** 2015-02-09

**Authors:** Yasuaki Tamaki, Takashi Nakayama, Kenichiro Kita, Katsutosi Miyatake, Yoshiteru Kawasaki, Koji Fujii, Yoshitsugu Takeda

**Affiliations:** Department of Orthopedic Surgery, Tokushima Red Cross Hospital, 103 Irinokuchi, Komatsushima-cho, Komatsushima, Tokushima 773-8502, Japan

## Abstract

Tension band wiring with cerclage wiring is most widely used for treating displaced patellar fractures. Although wire breakage is not uncommon, migration of a fragment of the broken wire is rare, especially migration into the knee joint. We describe here a rare case of migration of a wire fragment into the posterior septum of the knee joint after fixation of a displaced patellar fracture with tension band wiring and cerclage wiring. Although it was difficult to determine whether the wire fragment was located within or outside the knee joint from the preoperative plain radiographs or three-dimensional computed tomography (3D CT), we found it arthroscopically through the posterior transseptal portal with assistance of intraoperative fluoroscopy. Surgeons who treat such cases should bear in mind the possibility that wire could be embedded in the posterior septum of the knee joint.

## 1. Introduction

Tension band wiring with cerclage wiring is a common procedure for treating displaced patellar fractures. Subsequent breakage of the wire is not uncommon, but migration of a fragment of the broken wire has not been well described. To our knowledge, six reports describe such migration following tension band or cerclage wiring for seven patellar fractures, and only two cases of intra-articular translocation of a wire fragment have been documented [1–6].

Here, we present a rare case of wire fragment migration into the posterior septum of the knee after the tension band wiring with cerclage wiring for a comminuted patellar fracture. The case highlights that fragments can become embedded in the posterior septum of the knee joint. If treating surgeons do not know this, they might experience difficulty finding the fragment arthroscopically and possibly change the approach from the posterior aspect of the knee.

The patient was informed that data concerning this case would be submitted for publication.

## 2. Case Presentation

**Figure 1:**
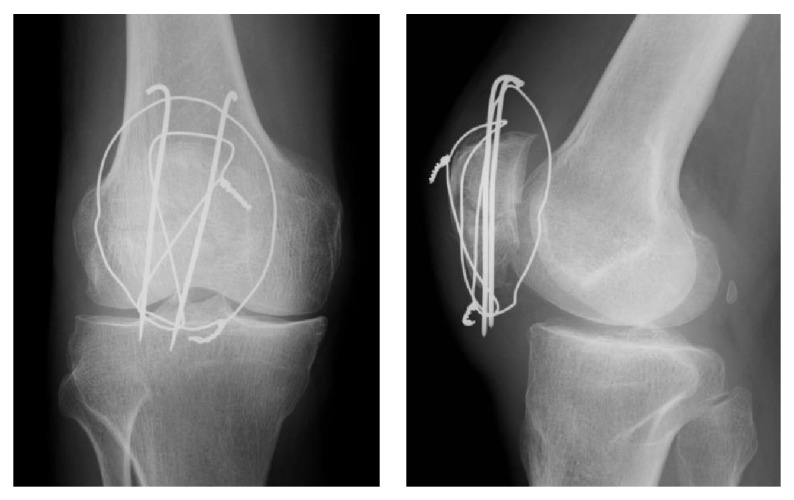
Plain radiographs after the primary surgery.

**Figure 2:**
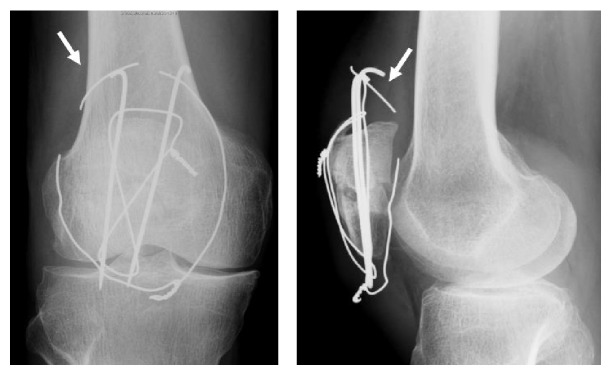
Plain radiographs at 10 weeks postoperatively show breakage of the superior portion of the cerclage wire (arrow).

A 75-year-old man underwent tension band wiring with cerclage wiring for treatment of a transverse patellar fracture of the right knee (Figure 1). At 10 weeks postoperatively, radiographs demonstrated breakage of the superior portion of the cerclage wire (Figure 2). Although he was advised to have all fixation devices removed after fracture healing and continuous follow-up, the patient was lost to follow-up after a clinical examination and plain radiography at 16 weeks postoperatively because he had no symptoms such as irritation due to the wire or limitation of range of motion. At 16 weeks, the wire was still radiographically at the superior part of the right patella.

**Figure 3:**
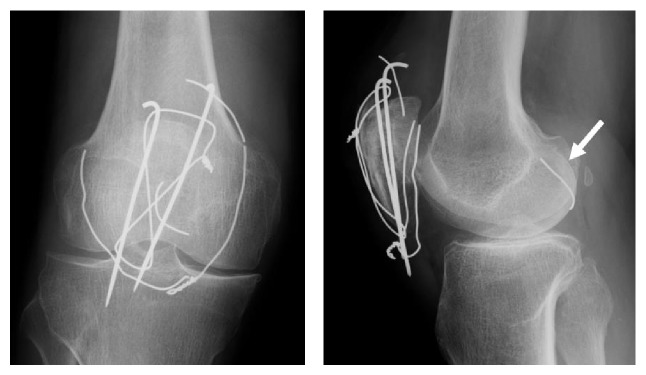
Plain radiographs of the right knee show a fragment of wire migrated into the posterior compartment of the right knee (arrow).

**Figure 4:**
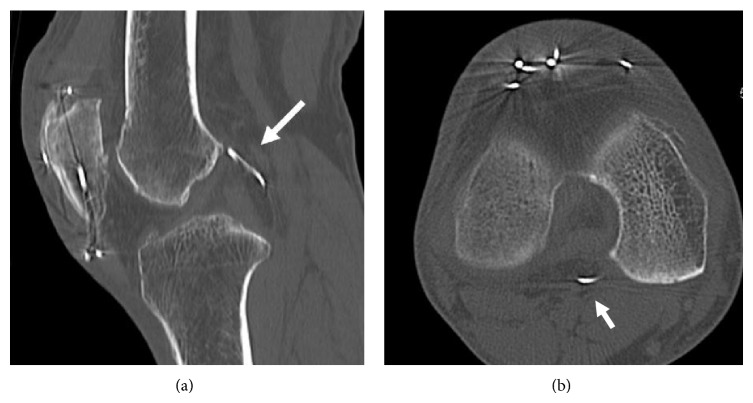
Computed tomography, (a) sagittal and (b) axial views. We could not determine from the scan whether the wire fragment was in the posterior compartment of the knee joint or popliteal fossa.

The patient returned to our outpatient clinic 2 years after the surgery because the wire had perforated the skin anteriorly. He did not recall any trauma to the knee since the last follow-up. Radiographs of the right knee demonstrated that the cerclage wire was broken into pieces, and a fragment had migrated into the posterior compartment (Figure 3). We could not decide from plain radiographs or three-dimensional computed tomography (3D CT) whether the wire fragment was in the posterior compartment of the knee joint or the popliteal fossa (Figure 4). Because there was a risk of chondral damage if it was inside the joint or neurovascular damage if it was in the popliteal fossa, the patient agreed to surgery to remove the hardware.

**Figure 5:**
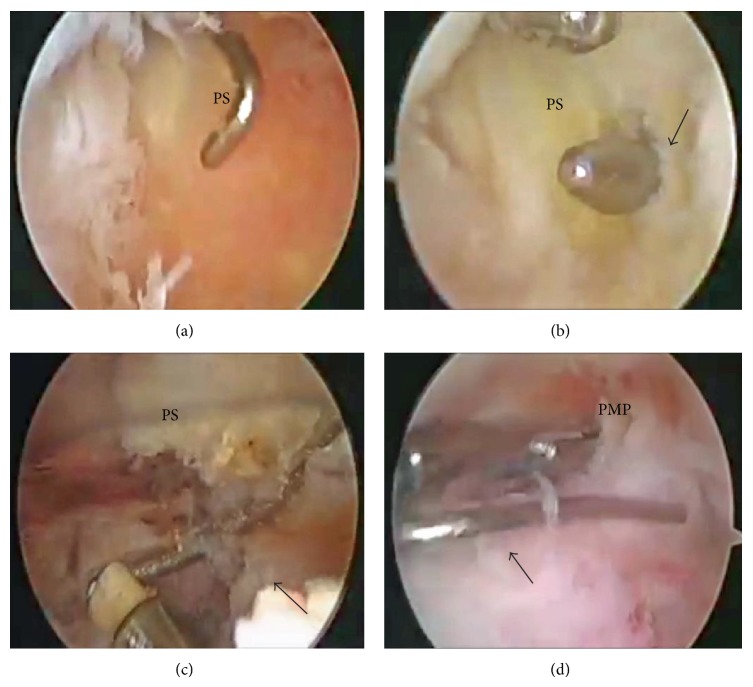
(a) Arthroscopic view from the posteromedial portal shows the palpation of the posterior septum (PS) by an arthroscopic probe through the anteromedial portal. (b) Arthroscopic view from the posteromedial portal shows a small hole (the posterior transseptal portal) (arrow) made at the central portion of posterior septum (PS) behind the PCL. (c) The broken wire (arrow) embedded in the posterior septum (PS) was exposed by enlarging the transseptal portal. (d) Arthroscopic view from the anteromedial portal shows the removal of the broken wire (arrow) through the posteromedial portal (PMP).

**Figure 6:**
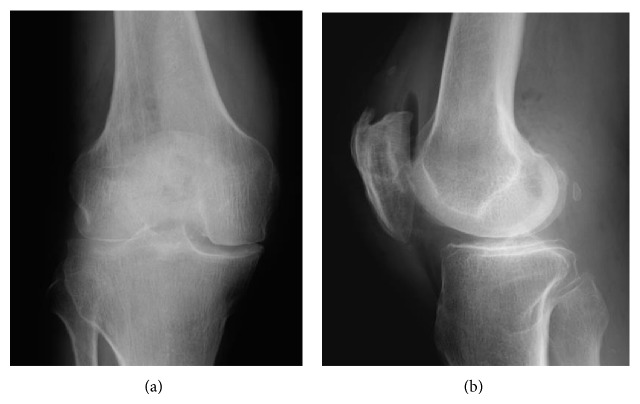
Plain radiographs after surgery to remove all wires.

A skin incision was made anteriorly over the previous incision and all the wires were removed. An arthroscopic examination was then performed; before scoping the posterior compartment, we performed the routine arthroscopic assessment through the standard anteromedial portal. Any structural damage including chondral lesion due to the wire migration was not found. Then, we examined the posterior compartment through the posteromedial or posterolateral portal. However, we could not find the wire fragment by palpating with a probe. However, we could not find the wire fragment by palpating the posterior structures with a probe introduced through the posteromedial or posterolateral portal. Before finishing the arthroscopic examination and approaching the popliteal fossa from the posterior aspect of the knee, we used intraoperative fluoroscopy, which demonstrated that the wire fragment had moved when we palpated the upper part of the posterior septum with the arthroscopic probe. This suggested that the wire was embedded in the posterior septum. We therefore made a posterior transseptal portal according to the procedure of Ahn and Ha and enlarged the portal upwards with a radiofrequency probe [7]. We eventually found the fragment embedded in the septum and were able to remove it safely through the posteromedial portal (Figures 5 and 6).

After surgery, the patient could walk without pain and showed full range of motion at final follow-up.

## 3. Discussion

The literature mentions very few cases of wire fragment migration. Those reported describe migration from the patella to the tibia, knee joint, posterolateral or posterior aspect of the knee, and the right ventricle of the heart [1–6, 8]. But the present case is the first of migration to the posterior septum of the knee joint. There are two possible paths the fragment took from the patella to the posterior septum: (1) the fragment migrated along the subretinacular layer to the popliteal fossa and then entered the posterior septum by penetrating the posterior capsule or (2) the fragment penetrated the anterior capsule into the knee joint and moved to the posterior compartment, becoming embedded in the posterior septum. Similar suggestions have been previously reported. Meena et al. suggested that pieces of broken K-wires found embedded under the quadriceps tendon had migrated along the subretinacular layer to the posterolateral popliteal fossa under the condition of repeated knee motion [5]. Choi et al. reported fragments of K-wires migrating to the popliteal fossa in two cases [1]. Wang and Lee described a case of intra-articular migration with nonunion of a patella fracture [4], speculating that the fragment migrated across the pseudoarthrosis line into the joint, because it would be difficult, in the absence of this fracture gap, for the wire to find an intra-articular route as this would require the wire to pierce through a considerable amount of soft tissue before entering the knee joint. However, Chen et al. did encounter a case of intra-articular migration without nonunion [3] and hypothesized that the migration occurred because the placement of the cerclage wire around the patella was too posterior. They speculated that when this wire broke, there was little to no posterior soft-tissue buttress, and the wire fragment easily penetrated through the joint capsule. In our case, we saw no chondral damage inside the joint, suggesting a path from the popliteal fossa was more likely; however, the actual path remains unclear.

Before the surgery, we could not determine whether the wire fragment was inside the joint or in the popliteal fossa based on examination of the plain radiographs and 3D CT images. Therefore, we planned to approach the knee joint first arthroscopically; then if we could not find the fragment inside the joint, we would turn the patient into the prone position and approach from the posterior. During the arthroscopic examination, we meticulously examined the posterior compartment with careful probing through the posterolateral and posteromedial portals but could not find the fragment. We almost turned the patient into the prone position, but the intraoperative fluoroscopy helped us to avoid this extra procedure. Once we knew that it was embedded in the posterior septum, we found the wire easily and safely using the posterior transseptal portal developed by Ahn and Ha [7].

To reduce the risk of complications related to conventional tension band wiring using a K-wire and metal wires, several novel techniques have been developed. Berg proposed the technique of tension band wiring through cannulated screws instead of K-wires [9]. Qi et al. developed this technique by using bioabsorbable cannulated screws with a braided polyester suture [10]. Yotsumoto et al. treated transverse patellar fractures using metallic ring pins and braided polyblend sutures [11]. They recommended this technique to reduce incidence of complications related to metallic materials, as the ring pins and braided polyblend suture do not migrate from the reduction site, being self-locking. Although a lower complication rate was reported using these techniques, the conventional tension band wiring described by the AO Foundation is still the most widely used. Thus, careful long-term follow-up is advised, and once the breakage of hardware is observed, the patient should be strongly advised to have the hardware removed. However, it is not uncommon that patients with hardware breakage refuse to have the surgery when it is asymptomatic. In fact, our case was lost to follow-up in spite of our advice for hardware removal after fracture healing and did not return to us until the broken wire penetrated the skin. He grudgingly consented to undergo the removal of the broken wire after he has been informed of the high risk of neurovascular or chondral damage. Surgeons who treat patients with broken wires should explain to their patients the possibility of critical complications, because they can migrate to the neurovascular bundles in the popliteal fossa [1, 5] and to the heart via the venous system [2].

We reported a case of migration of a broken wire into the posterior septum of the knee joint. Although it is extremely rare, when the preoperative images demonstrate the migrated wire is in the posterior part of the knee, which especially described the migrated wire in front of popliteal artery, surgeons should bear in mind the possibility of the wire in the posterior septum. If such is the case, the wire may be found safely through the posterior transseptal portal with assistance of intraoperative fluoroscopy.
